# An Update to the SBML Human-Readable Antimony Language

**Published:** 2024-05-23

**Authors:** Lucian Smith, Herbert M. Sauro

**Affiliations:** Department of Bioengineering, University of Washington, Seattle, WA 98195-5061; Department of Bioengineering, University of Washington, Seattle, WA 98195-5061

**Keywords:** Antimony, SBML, Systems Biology

## Abstract

Antimony is a high-level, human-readable text-based language designed for defining and sharing models in the systems biology community. It enables scientists to describe biochemical networks and systems using a simple and intuitive syntax. It allows users to easily create, modify, and distribute reproducible computational models. By allowing the concise representation of complex biological processes, Antimony enhances collaborative efforts, improves reproducibility, and accelerates the iterative development of models in systems biology. This paper provides an update to the Antimony language since it was introduced in 2009. In particular, we highlight new annotation features, support for flux balance analysis, a new rateOf method, support for probability distributions and uncertainty, named stochiometries, and algebraic rules. Antimony is also now distributed as a C/C++ library, together with python and Julia bindings, as well as a JavaScript version for use within a web browser. Availability: https://github.com/sys-bio/antimony.

## 1 Introduction

Antimony is a high-level, human-readable text-based language designed for defining and sharing models in the systems biology community. We developed it as a human-readable format to complement the XML-based SBML(Hucka et al., 2003) standard. In general, Antimony is converted to SBML which can then be loaded into the many compatible SBML simulators such as libroadrunner (Welsh et al., 2023). Often simulators will convert the SBML into a set of differential equations which are then solved by the simulator to generate time course data.

Antimony uses a minimum of punctuation characters which renders the text easier to read and understand. Antimony is implemented using C/C++ (Smith et al., 2009) and Bison as the grammar parser. However, the distribution also includes Python bindings which can be installed using pip to make it easy to use from Python. Julia bindings are also available to users. It is also available via the Tellurium package (Choi et al., 2018; Medley et al., 2018) and more recently a JavaScript/WASM version was created which allows the Antimony language to be used on the web. The website tool makesbml uses the Javascript version (Jardine, Smith and Sauro, 2023) generated from emscripten (https://emscripten.org/). There is also a pure Javascript parser for the Antimony web editor (https://github.com/sys-bio/AntimonyEditor)

Antimony supports SBML Level 3, version 2, and the following SBML packages: Hierarchical Model Composition (Smith et al., 2015), Flux Balance Constraints (Olivier and Bergmann, 2015) and Distributions (Smith et al., 2020).

In this article, we will describe some of the major updates that have been incorporated into Antimony since its inception.

## 2 Examples

In this section, we will show some examples of basic Antimony functionality that doesn’t include the new features. The first example is a single chemical reaction whose rate is governed by simple mass-action kinetics:


// Define a single reaction with reactant A and product B and rate law k1*A
A −> B; k1*A
// Initialize values
A = 5 
B = 0 
k1 = 0.1


Listing 1: Antimony model of a single reaction

Multiple reactions can be included in a model. For example, here is a simple branched pathway where the third reaction uses a reversible mass-action rate law.


// Define three reactions forming a simple branch
A −> B; k1*A
B −> C; k2*B
B −> D; k3*B - k4*C
// Initialize values
A = 5; B = 0; C = 0; D = 0;
k1 = 0.1; k2 = 0.2; k3 = 0.15; k4 = 3.4


Listing 2: Antimony model of a simple branched pathway

Multiple reaction/product reactions with non-unity stoichiometries are also easily specified as follows:


// A reaction with multiple reactants
A + B −> C; k1*A*B
// A reaction with non-unity stoichiometry
2 C −> D + E; k2*C
// Initialize values
A = 5; B = 0; C = 0; D = 0; E = 0; k1 = 0.1; k2 = 0.2;


Listing 3: Antimony model using non-unity stoichiometries

Named reactions can also be specified as follows. A simulator may use the reaction name to request values for the reaction rate:


// Name a reaction ‘Jl’.
J1: A + B −> C; k1*A*B
// Initialize values
A = 5; B = 2; C = 0; k1 = 0.1;


Listing 4: Antimony model using named reactions

Another example shows how external species or species that are determined by external forcing functions (known as assignment rules in SBML) can be described. External species (or boundary species in SBML terminology) are by default fixed. In practice, a simulator will not generate a differential equation for such species.


// A reaction with an external reactant, A, indicated with a dollar symbol.
// Species A will be fixed in any model generated from this script 
$A −> B; k1*A
// A reaction with a forcing function. In this case, the function
// is a sine wave of a given frequency and amplitude. Note the use of
// the ‘:=‘ assignment symbol to indicate this equation is part of the model
// and will be executed continuously when the model is simulated
X := 1 + amp*sin (time*freq)
$X −> Y; k1*X
// Initialize values
A = 1
amp = 2.5; freq = 0.5; k1 = 0.1;


Listing 5: Antimony model using a fixed species and a forcing function.

Rates of change can also be set directly: *dS/dt* in Antimony is represented as *S*^′^. When declaring a rate of change for a species that appears in other reactions, that species must be declared as a boundary species, much like a species with a forcing function.


// Define a reaction.
J1: $A + B −> C; k1*A*B
// The ‘A’ species here is not consumed by reaction J1, and instead increases
// at an autocatalytic rate
$A’ = k2 * A
// Initialize values
A = 5; B = 2; C = 0; k1 = 0.1; k2 = 0.01


Listing 6: Antimony model using explicit rates of change

Other useful features include the ability to define discrete events based on a boolean expression, the ability to build modular models that can be combined to create a larger model. The documentation for these as well as additional features can be found at (https://tellurium.readthedocs.io/en/latest/antimony.html).

In the following sections, we will describe several significant enhancements that have been made to the Antimony language since its inception.

## 3 Metadata Extensions

The BioModels repository Malik-Sheriff et al. (2020) contains a large number of curated models. In almost all cases, the curators at BioModels have added useful metadata to the model such as the author of the model, a link to the paper etc. Antimony has been extended to allow users to easily add metadata to a model or when retrieving a model in SBML the metadata is shown in the Antimony model. The following is an example of metadata being added to a model using Antimony:


model model_source “http://identifiers.org/biomodels.db/BIOMD0000000141”
model publication “http://identifiers.org/pubmed/15484883“ 
model biological_system “http://identifiers.org/go/GO:0019228”
model taxon “http://identifiers.org/taxonomy/40674”
model created “2007–07-16T09:41:14Z” 
model modified “2015–02-25T11:18:57Z” 
model creator1.givenName “John”
model creator1.familyName “Smith” 
model creator1.organization “Oxford” 
model creator1.email “John@university.edu“ 
model notes “This model describes how...”


Note that when using the metadata feature, a model must be bracketed by model name() and end. A simple example below illustrates this requirement:


model mymodel() 
S1 −> S2; k1*S1
model notes “This is a small model” 
end


Listing 7: Antimony model using a fixed species and a forcing function.

## 4 rateOf

rateOf is a function call that can be used to retrieve the rate of change of a species or a rate rule. It can not be used to obtain the rate of change of a reaction rate or the left-hand side of an assignment rule. An example is given below which illustrates how to obtain the rate of change of a species S1 or the value of a differential equation, C’ = 10*k3. The rateOf method can also be inserted into an equation, such as a reaction rate equation, assignment rules, etc.


J0: S0 −> S1; k0*S0
J1: S1 −> S2; k1*S1
X1 := rateOf(S1)
C’ = 10*k3
X2 := rateOf(C)


Listing 8: Retrieving the rare of change of a species or rate rule.

## 5 Probability Distributions

The latest version of Antimony allows a modeler to initialize values drawn from a given distribution function. For example, in the code below, S0 and S1 are drawn from a normal distribution with mean 0.5 and a standard deviation 0.2. This can be useful for example when investigating how uncertainties propagate through a model. Repeated runs of such a model will result in variations in the outputs.

The distribution functions can also be used in the outcome of an event. For example, if an event is used to detect when a species passes a certain value, we can change a rate constant based on a distribution. In the example below, if S1 exceeds 10, we set the rate constant k0 based on a uniform distribution between 2.6 and 5.5. Note that distribution may only be used in discrete contexts (such as initializing a value or resetting a value from an event) and cannot be used in continuous contexts such as in reaction rates or assignment rules.


J0: S0 −> S1; k0*S0 
J1: S1 −> S2; k1*S1
if S1 > 10: 
k0 = uniform(2.5, 5.5)
S0 = normal(0.5, 0.2)
S1 = normal(0.5, 0.2)
S2 = 0


Listing 9: Initializing values based on a probability distribution.

A variety of distributions are provided and are shown in the table below. Details of these distributions can be found in the SBML distribution documentation Smith et al. (2020).


normal(mean, stddev) 
normal(mean, stddev, min, max) 
uniform(min, max) 
bernoulli(prob)
binomial(nTrials, probabilityOfSuccess) 
binomial(nTrials, probabilityOfSuccess, min, max) 
cauchy(location, scale) 
cauchy(location, scale, min, max) 
chisquare(degreesOfFreedom) 
chisquare(degreesOfFreedom, min, max)
exponential(rate) 
exponential(rate, min, max) 
gamma(shape, scale) 
gamma(shape, scale, min, max) 
laplace(location, scale) 
laplace(location, scale, min, max) 
lognormal(mean, stdev) 
lognormal(mean, stdev, min, max)
poisson(rate) 
poisson(rate, min, max) 
rayleigh(scale) 
rayleigh(scale, min, max)


## 6 Uncertainty

The SBML ‘Distributions’ package introduced a variety of ways to store information about the uncertainty of model elements. Antimony has now been extended to store this same information through the following syntax:


A.mean = x
A.stdev = x (or A.standardDeviation = x)
A.coefficientOfVariation = x
A.kurtosis = x
A.median = x
A.mode = x
A.sampleSize = x
A.skewness = x
A.standardError = x
A.variance = x
A.confidenceInterval = {x, y}
A.credibleInterval = {x, y}
A.interquartileRange = {x,y}
A.range = {x,y}
A.distribution = function()
A.distribution is “http://uri“
A.externalParameter = x || {x,y} || function()
A.externalParameter is “http://uri“


Listing 10: Uncertainty information attached to variables.

In the above examples, A may be any symbol in Antimony with mathematical meaning; x and y may be either a symbol or a value (i.e. A.mean=2.4; A.confidenceInterval={S1, 8.2}); function() may be any mathematical formula; and “http://uri“ is a URI that defines the given distribution or externalParameter.

## 7 Flux Balance Constraints

The SBML ‘Flux Balance Constraints’ (‘FBC’) package introduced a way to define a system of reactions, each constrained in different ways, together with an objective function to be maximized or minimized. In this way, some conclusions may be reached about the fluxes in a reaction network without knowing the specifics of the reaction rate equations. Antimony has now been extended to store this information through the following syntax:


// The objective function (for FBC analysis): 
OBJF: maximize R16 + R03;
// Constraints:
constraint c0: R16 >= 0 
constraint c1: R16 <= 1000 
constraint c2: R03 >= −1000 
constraint c3: R03 <= 1000 
constraint c4: R02 >= −1000 
constraint c5: R02 <= 1000


Listing 11: Flux balance constraint information.

In the above example, an objective function OBJF has been defined that simply states that the sum of the reaction rates of R16 and R03 should be as large as possible (the reaction network itself is not shown here; there are often many reaction defined, none of which have reaction rates). Some example constraints are listed, collectively stating that reaction R16 must be between 0 and 1000, with R03 and R02 constrained to be between −1000 and 1000. In a complete model, every reaction would have a lower and upper bound (though it may be positive or negative infinity).

Note that Antimony is currently only compatible with FBC version 1, so models encoded using other versions of the FBC package are translated to version 1 before importing into Antimony. Some information introduced in later versions of FBC may be lost, though we intend to update FBC support to include that extra information.

## 8 Named Stoichiometries

SBML permits reaction stoichiometries to be set using variables. An example is given below where the second reaction has stoichiometries n and m. These can be set using either an assignment rule or during initializing. The latter can be useful when comparing how a simulation responds to different stoichiometries. A good example might be investigating how a reaction rate responds to different stoichiometries, such as with a Hill-type mechanism.

Changing the stoichiometry using a simulation by using an assignment rule, is more problematic and depends on how the simulator implements this functionality. The problem is that the model is being physically changed during the simulation which requires careful management of the differential equation solver.


J0: S0 −> S1; k0*S0 
J1: n S1 −> m S2; k1*S1^n
n := k2/2 
m = 2/3


Listing 12: Setting stoichiometries using variables.

## 9 Algebraic Rules

Algebraic rules are defined as equations that are always true but do not declare which variable or variables should be adjusted to ensure that they are true. Algebraic rules have somewhat limited support in some simulators, and are not, for example, supported by Tellurium. They are provided by Antimony solely for users who wish to export the model and use it in other systems that do support such functionality.

To declare an algebraic rule, optionally give it a name, and then declare “0 = [formula]”:


0 = S1*k1 – 10 
alg2: 0 = S2*k2 – 20


Listing 13: Examples of algebraic laws using Antimony.

## 10 Availability

Antimony is a reusable C/C++ library which means that other applications can host Antimony which will allow the host application to easily import and export SBML via the Antimony language. Source code and binary releases for all platforms are available at (https://github.com/sys-bio/antimony). The library itself has a small standard C API making it easy to link to any computer language. For example, Python bindings are available via a pip package (https://pypi.org/project/antimony/). A QT-based application called QTAntimony provides a graphical user interface that makes it easy to edit Antimony and translate to and from SBML. Julia bindings Welsh et al. (2023) are available as well as a JavaScript library that can be used from within a web browser Jardine, Smith and Sauro (2023).

The source code is licensed under the liberal BSD-3 license, making it suitable for commercial as well as academic use.

## 11 Future Updates

There are several future updates we wish to implement. The most significant is to implement a human-readable extension for the SBML layout/render packages. Layout Gauges et al. (2006) permits users to specify the visual layout of a biochemical network model described in the SBML. Render Bergmann et al. (2018) is an extension to the layout package that permits users to specify the look and feel of a laid-out network, in terms of colors, line thickness etc.
